# Effectiveness and Cost-Effectiveness of a Mobile Psychoeducation Program (OkeyMind) for Social Anxiety Symptoms Among Youths: Protocol for a Randomized Controlled Trial

**DOI:** 10.2196/64518

**Published:** 2025-07-14

**Authors:** Yi-Zhou Wang, De-Hui Ruth Zhou, Siu-Man Ng, Bei-Bei Wang, Yu-Ya Feng, Xue Weng

**Affiliations:** 1 Department of Counselling and Psychology Hong Kong Shue Yan University Hong Kong SAR China (Hong Kong); 2 Department of Social Work and Social Administration University of Hong Kong Hong Kong SAR China (Hong Kong); 3 School of Education Xin Yang University Henan China; 4 Center of Psychological Health Education Suzhou Institute of Technology Jiangsu China; 5 Institute of Advanced Studies in Humanities and Social Sciences Beijing Normal University Zhuhai China

**Keywords:** mHealth, digital health, Chinese, low cost, youth friendly

## Abstract

**Background:**

Social anxiety is increasingly prevalent among youths, leading to social withdrawal, isolation, and heightened depression risk. Mobile health (mHealth) interventions offer anonymity, accessibility, and personalized support, but their effectiveness and cost-effectiveness for young individuals with social anxiety remain unclear.

**Objective:**

This randomized controlled trial aims to evaluate the effectiveness and cost-effectiveness of a mobile-based psychoeducation program (OkeyMind) for mitigating social anxiety symptoms among youth.

**Methods:**

Participants aged 15 years through 24 years with mild or more severe social phobia (Social Phobia Inventory [SPIN] ≥25) and access to WeChat will be recruited and screened onsite and then randomly assigned to the intervention or waiting list control group. The study will recruit 180 participants, with 90 in each group. The intervention group will receive a 1-month mobile psychoeducation program (OkeyMind), which includes both psychoeducation and meditation components, with all content fully automated. The waiting list control group will receive the same intervention after 3 months. The primary outcome is social anxiety symptoms measured using SPIN. Secondary outcomes include depression (Patient Health Questionnaire-9) and health-related quality of life (EuroQol-5). Assessments will be conducted at baseline, postintervention, and a 3-month follow-up.

**Results:**

Recruitment began in September 2024. The study was funded in April 2024, and the first participant was enrolled in September 2024; as of the end of March 2025, 132 participants had been recruited. Recruitment is ongoing. Data collection will conclude in July 2025, after which data analysis will begin and be completed in August 2025, with primary findings targeted for publication in spring 2026.

**Conclusions:**

This research evaluates the effectiveness and cost-effectiveness of OkeyMind, a mobile-based psychoeducation program, for reducing social anxiety symptoms among youth. OkeyMind might be a promising strategy for combating social anxiety, especially in low- and middle-income countries with limited medical resources. If effective, this intervention could provide an accessible, cost-effective approach to managing social anxiety, particularly in resource-limited settings.

**Trial Registration:**

ClinicalTrials.gov NCT06490315; https://clinicaltrials.gov/study/NCT06490315

**International Registered Report Identifier (IRRID):**

PRR1-10.2196/64518

## Introduction

### Social Anxiety Among Youths

Social anxiety, also known as social phobia, is characterized by a marked fear of social situations where individuals may be scrutinized by others, often leading to avoidance behaviors and distress [1,2]. A core component of social anxiety is heightened sensitivity to negative evaluation, reinforcing self-doubt and anticipatory anxiety [1]. This persistent self-doubt and anticipatory anxiety can lead to social withdrawal, reducing social support and reinforcing feelings of isolation. Over time, these factors contribute to a negative self-concept and sense of helplessness, both of which are strongly associated with depression [3]. Numerous studies have demonstrated the link between social anxiety and depression [3-5]. A study tracking 2548 participants aged 14 years through 24 years from diverse communities found that social anxiety frequently coexists with major depression and, in most cases, social anxiety develops first, preceding the onset of depression [6].

Moreover, a frequently overlooked aspect in academic discussions is the distinction in depression between youths and the general adult population. This difference is significant due to various factors. First, youths experience rapid physical, emotional, and cognitive development, making them more vulnerable to depression during this period of intense change [7]. Additionally, young individuals are still developing effective coping strategies, making them more susceptible to negative influences, whereas adults, with more life experience, generally have better-established coping mechanisms [8]. Furthermore, youths often depend on guardians for access to mental health care, which can be hindered by stigma or a lack of awareness, while adults have more autonomy in seeking professional help [9]. Therefore, there is a growing demand for interventions specifically designed to address social anxiety in youths, tailored to the distinct characteristics unique to this age group.

### mHealth Interventions as an Effective and Cost-Effective Method

Over the last decade, there has been a notable increase in the use of mobile health (mHealth) technologies to improve both access to and engagement with treatments for social anxiety [10]. Accumulated clinical evidence broadly supports this widespread promotion, especially in the young population. mHealth solutions in the delivery of interventions are widely accepted by youth users. Most young adults own a smartphone, have accessed mHealth apps, and report a stated preference for engaging with health services in this manner [11]. Moreover, mHealth interventions are capable of replicating, and even surpassing, the therapeutic gains achieved through traditional face-to-face treatments [12]. Studies indicate that these approaches are producing promising positive effects in reducing psychological distress, including symptoms of social anxiety [13-15]. The mobile intervention being evaluated in this study, OkeyMind, is designed to meet the specific needs of the target population and intended outcomes by leveraging WeChat’s extensive global user base of 1.382 billion active users [16]. It provides a highly accessible and lightweight alternative to traditional mobile apps by eliminating the need for additional downloads and storage. Additionally, OkeyMind incorporates innovative features, including personalized feedback mechanisms for tracking progress and adaptive algorithms that tailor the intervention to individual needs. Given that young users tend to place a high value on privacy, convenience, and customization, this approach ensures greater usability and relevance for the target population [17,18].

Moreover, the delivery of mHealth services has demonstrated significant cost efficiency, a conclusion strongly supported by recent meta-analyses and synthesis studies [19,20]. This is especially evident in the realm of mental health, where mHealth solutions stand out for their ability to eliminate the need for physical infrastructure [21]. OkeyMind, a WeChat miniprogram, has a lower development complexity and shorter implementation cycle than a standalone mobile app, as it operates within the WeChat ecosystem, eliminating the need for separate app store approvals and extensive backend infrastructure [22,23]. Therefore, OkeyMind has the potential to be more easily accessible to a wider audience, including individuals who may face barriers to traditional in-person therapy, such as geographical limitations, scheduling conflicts, or stigma associated with seeking help [24].

### Research Gaps

Despite the rising prevalence of social anxiety among youths, there is limited high-quality empirical evidence, particularly from randomized controlled trials, on effective interventions tailored to youth with social anxiety symptoms. mHealth interventions have shown promise, yet their efficacy remains underexplored [25]. Additionally, the scarcity of economic evaluations for psychological interventions becomes especially critical during global economic downturns, when funding for mental health support and research typically decreases [26]. The absence of economic evaluations further complicates the situation by not providing essential data on the interventions’ cost-effectiveness and benefits. Without this information, policymakers struggle to allocate limited resources effectively, risking the sustainability of mental health services and potentially increasing the unmet need for psychological support in populations affected by economic hardship [27].

### Research Objectives and Hypotheses

The primary objective is to assess the effectiveness of the OkeyMind mobile-based psychoeducation program on mitigating social anxiety symptoms among young adults aged 15 years through 24 years. The secondary objective is to evaluate the cost-effectiveness of the OkeyMind program as a mental health intervention tool, aiming to ascertain whether the financial investment in OkeyMind translates into significant economic and health-related advantages. The study tests 2 hypotheses: (1) OkeyMind is more effective than the control condition in reducing social anxiety symptoms among youths, and (2) OkeyMind is more cost-effective than the control condition for this population.

## Methods

### Trial Design

**Figure 1:**
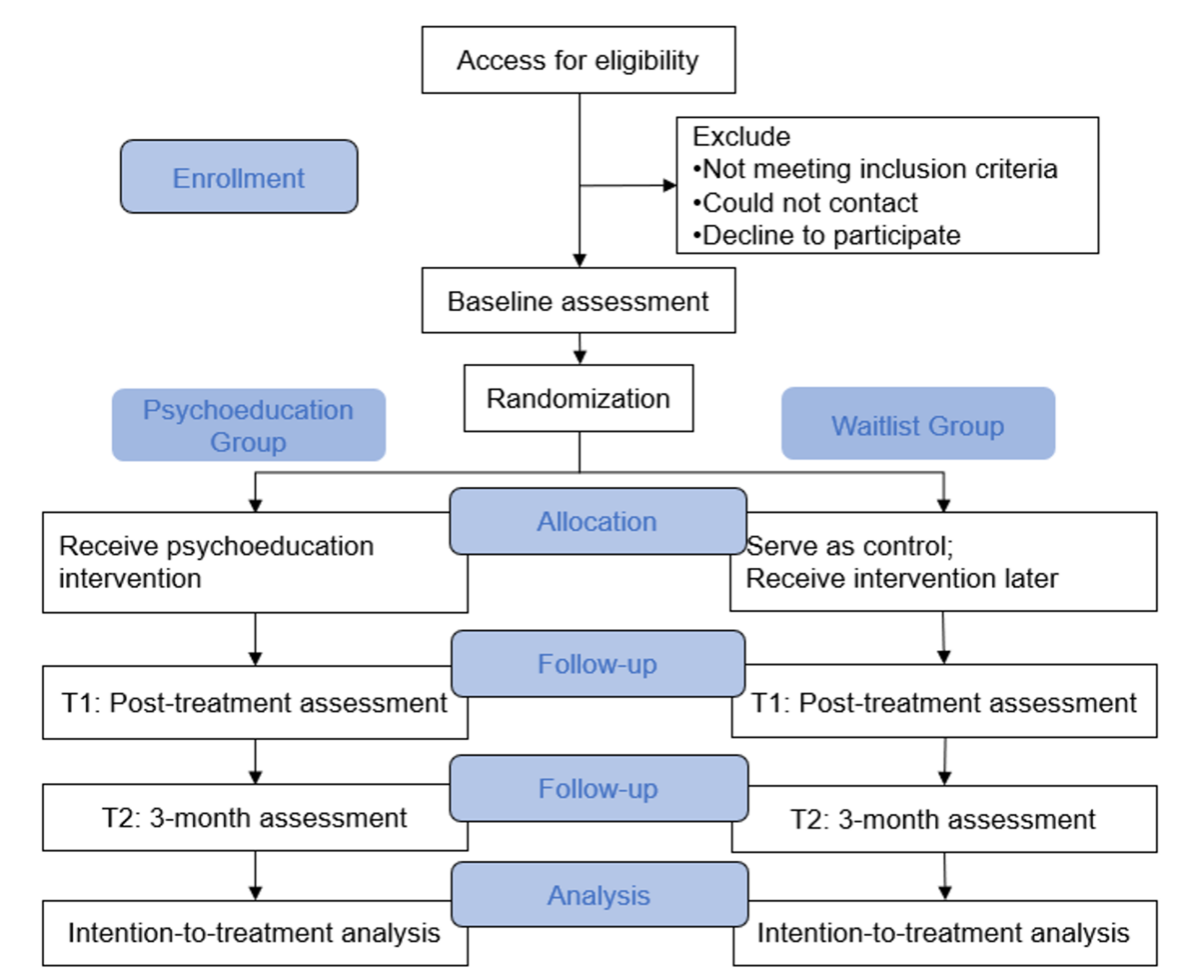
CONSORT (Consolidated Standards of Reporting Trials) flow diagram.

The design is a 2-arm, single-blind, parallel-group, randomized controlled trial to test whether the mHealth intervention program (OkeyMind), compared with the waiting list control, leads to reductions in social anxiety symptoms among youths. No additional interventions are provided beyond the OkeyMind program during the research period. Assessments will be carried out at baseline (T0), posttreatment (T1), and follow-up (T2). The choice of a 3-month follow-up assessment is primarily driven by its established feasibility in similar studies [24]. The protocol has been registered with ClinicalTrials.gov (NCT06490315). Figure 1 shows the CONSORT (Consolidated Standards of Reporting Trials) flow diagram.

### Ethical Considerations

The protocol and informed consent form received approval from the Hong Kong Shue Yan University of the Human Research Ethics Committee (HREC 24-06(6)). Eligible adult participants who are willing to join the study will provide consent by signing an online consent form. Eligible adolescent participants will be required to obtain parental consent before enrollment. To reduce the likelihood or detection of harm, various safety and security procedures will be implemented, such as excluding potentially psychologically ill participants during the screening stage.

To ensure the confidentiality and security of participant data, all collected information is securely encrypted during transmission and storage, preventing unauthorized access. Access to participant data is strictly limited to authorized research personnel. Additionally, personally identifiable information is anonymized or de-identified wherever possible to safeguard participant privacy. Each eligible participant will receive compensation of approximately HK$ 190 (US $25) for their participation in the study.

### Randomization and Blinding

Participants will be randomized after the baseline assessment using computer-generated block randomization with a 1:1 allocation ratio within pre-established blocks of 90 participants. To ensure balance, a randomized blocking schema with block sizes of 2, 4, or 6 will be implemented. An independent research assistant, uninvolved in statistical analysis, will conduct the randomization. To minimize bias, participants will remain blinded to their group assignment.

### Recruitment

The study will be conducted in school settings, including middle schools and tertiary institutions, across China. The trial aims to enroll 180 participants, with 90 assigned to each group [28]. This sample size accounts for an anticipated attrition rate of 10% and is expected to offer 90% statistical power. This determination is based on using an independent group *t* test, a significance level of .05, and an effect size of 0.35 (Cohen *d*), which falls within the small-to-medium range [29]. To recruit enough participants, school faculty help with recruitment, and digital flyers and promotional videos are shared through WeChat and school online platforms.

The participant inclusion criteria include (1) youths aged between 15 years and 24 years old who are (2) exhibiting mild or more severe social phobia (ie, Social Phobia Inventory [SPIN] score ≥25) and (3) have a smartphone with WeChat installed and basic internet literacy. The exclusion criteria include (1) being unable to attempt a baseline assessment (eg, due to being unpermitted to leave a psychiatric ward), (2) currently receiving another intensive psychological intervention, (3) currently holding an active suicidal plan, and (4) having a current diagnosis of psychological disorders (eg, schizophrenia, bipolar disorder). These data will be collected through self-report questionnaires, including questions such as: “Have you been previously diagnosed with any psychological disorders (eg, schizophrenia, bipolar disorder)? If yes, please specify.” Additionally, university tutors, who are familiar with students’ mental health conditions, will assist with identifying ineligible participants. Participants will be recruited and screened offline but will complete all interventions and assessments online. Participants are briefed through online tutorial video clips in the OkeyMind miniprogram; these clips demonstrate the research flow regarding intervention timing, follow-up frequency, and usage intensity.

### Protocol Development

#### Delivery Program

The intervention is delivered via a mobile phone using a WeChat miniprogram called OkeyMind. OkeyMind was initially tested during the COVID-19 pandemic in 2020. A pilot program was deployed to assess mental health conditions and promote mental health literacy among college students (n=254). Positive feedback from both students and staff emphasized the program’s usability, accessibility, and relevance. Insights from this pilot informed the development of OkeyMind, improving its user interface, engagement strategies, and intervention content, with a focus on social anxiety, while maintaining high completion and satisfaction rates.

OkeyMind differentiates itself from other mobile mental health interventions through 3 main features [30]: (1) targeted focus on social phobia, (2) innovative content delivery methods, and (3) integration of evidence-based mindfulness. OkeyMind is specifically designed to address social phobia, providing tailored psychoeducation and interventions that directly target social anxiety symptoms. To enhance engagement, OkeyMind uses cartoon-based video clips, influencer interviews, real-life cases from internet celebrities, and engaging animations, making psychoeducation more interactive and appealing to youth. This approach helps maintain attention and improves information retention compared with traditional lecture-style content. OkeyMind incorporates mindfulness practices developed by the Jockey Club “Peace and Awareness” Mindfulness Culture in Schools Initiative, which have been empirically shown to reduce stress, regulate emotions, and enhance overall well-being [31]. This evidence-based component strengthens the intervention’s effectiveness at supporting mental health.

OkeyMind features 3 main modules: self-learning module, psychological assessment tools, and meditation demonstrations. Participants can simply search “OkeyMind” on the WeChat miniprogram platform and access it free of charge. The mobile psychoeducation program (OkeyMind), based on the WeChat miniprogram, includes both psychoeducation and meditation components. Participants will complete the components as well as the psychological assessment tools at their own pace. For meditation practices, a tutorial video will be provided for guidance. All content is fully automated. No co-intervention will be provided alongside OkeyMind. To prevent delays in the intervention or follow-up assessments, an automated reminder function was developed. This feature ensures that participants receive pop-up reminders via WeChat if any delays occur. Trained research assistants will remind participants to engage in the activities (eg, intervention sessions, assessments) according to the study timeline and will be available to answer questions via social media platforms (eg, WeChat), phone calls, or messages.

#### Intervention Group—OkeyMind

This system features 3 main modules: self-learning modules, psychological assessment tools, and meditation demonstrations.

#### Self-Learning Modules

To design an appealing psychoeducation component for young adults, self-learning modules feature cartoon-based video clips, interviews with popular influencers, real-life cases from internet celebrities, and engaging animations. These entertaining and lively formats provide insights into social anxiety, educating users on the condition’s characteristics, coping mechanisms, and guidance for seeking professional assistance. By rejecting dull content and incorporating elements that resonate with youth, these resources aim to effectively educate and support young adults.

#### Psychological Assessment Tools

The program incorporates interactive, user-friendly tools designed to evaluate and monitor the youth using the program. These tools might include questionnaires and scales that assess levels of social anxiety, depression, and other relevant psychological states. Tailored to provide immediate feedback, the assessments are structured to be engaging, ensuring users are motivated to complete them. By analyzing responses, the program can offer personalized insights and track progress over time, allowing users to understand their mental health patterns better. This feature aids with not only self-awareness but also identifying areas where intervention might be needed, enhancing the overall effectiveness of the psychoeducation provided.

#### Meditation Demonstration

The program features guided meditation sessions led by calming, animated figures designed to appeal to young adults. These sessions aim to introduce users to various meditation techniques that can help manage symptoms of social anxiety. Each demonstration focuses on a specific meditation practice, such as mindfulness or breathing exercises, offering step-by-step guidance in a visually engaging and easy-to-follow manner. Through these demonstrations, users can learn and practice meditation in a way that is both informative and soothing, fostering skills that contribute to emotional and mental well-being.

Additionally, OkeyMind incorporates several strategies to improve adherence to intervention protocols and implements procedures for monitoring compliance, including (1) progress tracking, (2) reminders and notifications, and (3) optimal content length. Progress tracking includes features to monitor users’ progress over time, such as module completion. This functionality not only encourage continued engagement but also helps users see tangible signs of their journey and growth. The reminders and notifications use automated prompts to encourage consistent use of the app’s features and adherence to prescribed self-help activities. These reminders can play a critical role in fostering a routine and ensuring the beneficial use of the app. To ensure optimal content length, each video clip should be around 3 minutes to 5 minutes long, concise enough to maintain attention while providing substantial information or insights. Furthermore, complex concepts are broken down into digestible parts, with each video focusing on a single topic or coping strategy. This modular approach allows users to progress at their own pace.

#### Control Group—Waiting List

Participants in the waiting list control group will have access to the OkeyMind intervention withheld until after the 3-month follow-up assessment. To prevent confounding effects from relevant concomitant care and interventions, participants will be required to refrain from receiving any other treatments or interventions specifically targeting social phobia during the study period.

### Outcomes

**Figure 2:**
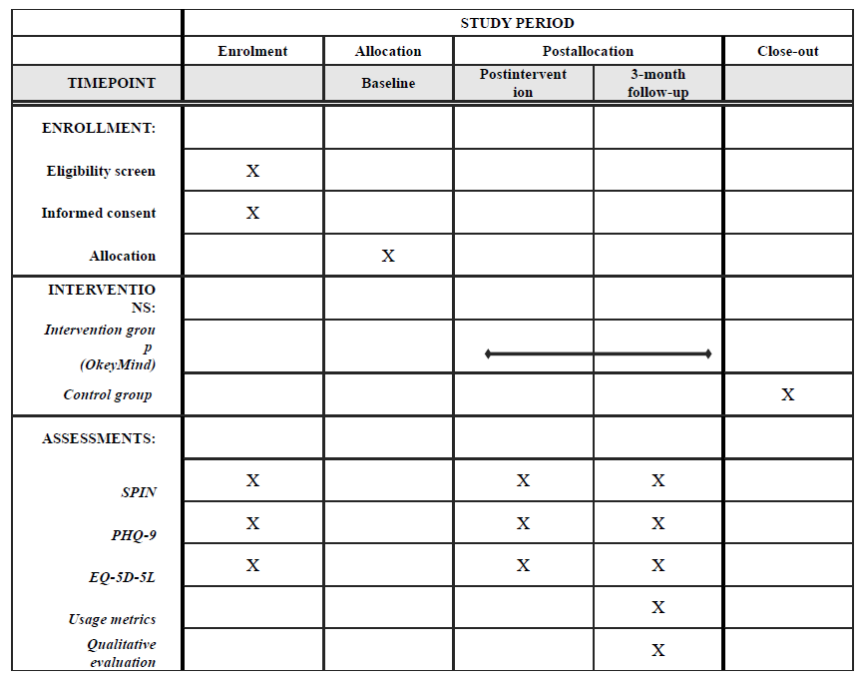
SPIRIT (Standard Protocol Items: Recommendations for Interventional Trials) checklist. PHQ-9: Patient Health Questionnaire-9; SPIN: Social Phobia Inventory.

Basic demographic and clinical data, including age, gender, region, and socioeconomic status, will be collected. The primary outcome will be social anxiety symptoms, assessed using SPIN. Secondary outcomes include depressive symptoms, measured using the Patient Health Questionnaire-9 (PHQ-9), and quality of life, evaluated using the EQ-5D-5L, which will also be used for cost analysis. All outcomes will be self-assessed using online measurements at baseline, postintervention, and the 3-month follow-up (Figure 2).

The primary outcome is social anxiety symptoms measured using SPIN. Developed by Antony et al in 2006 [32], SPIN is a brief self-report tool consisting of 17 items that assess fear, avoidance, and physiological discomfort in social situations. It is specifically designed for use with social anxiety disorder [32]. The Chinese version of the SPIN has been examined with good internal consistency (Cronbach α=0.85) and test-retest reliability (r=0.73). A cutoff value of 25 or higher has demonstrated strong reliability and validity and is commonly used to screen for social anxiety in the Chinese population [33].

Secondary outcomes include depressive symptoms measured using the PHQ-9 and quality of life measured using the EQ-5D-5L. The PHQ-9 is widely used to screen for the presence and severity of depression, monitor the severity of depression over time, and assist with the diagnosis of depression. Each of the 9 items is scored from 0 (not at all) to 3 (nearly every day), with a total score ranging from 0 to 27. Higher scores indicate more severe depressive symptoms. PHQ-9 scores of 5, 10, 15, and 20 represent mild, moderate, moderately severe, and severe depression, respectively. The Chinese version of the PHQ-9 demonstrates high internal validity and consistency, evidenced by a Cronbach α of 0.86 and strong test-retest reliability with a correlation coefficient of 0.86, affirming its efficacy for use in Chinese-speaking populations [34].

EQ-5D-5L is an established tool for assessing the general health status of individuals across a wide array of health conditions and treatments [35]. It measures health outcomes in 5 dimensions: mobility, self-care, usual activities, pain and discomfort, and anxiety and depression. Each dimension is rated on a 5-level scale, from no problems (level 1) to extreme problems (level 5), facilitating a comprehensive evaluation of a patient’s health-related quality of life. The total score is derived by converting the individual dimension scores into a single summary index value, reflecting overall health status, with higher scores indicating better health outcomes. Research supports the EQ-5D-5L’s reliability and validity among Chinese-speaking populations, showing good internal consistency with a Cronbach α between 0.76 and 0.79 [36-38].

To assess participant adherence, usage metrics will be analyzed using log files. This includes the number of video views, instances of video interruptions, intervention completion dates, and assessment completion dates. Completion rates will be classified as follows: ≥81% indicates sufficient engagement, 61%-80% indicates moderate engagement, 41%-60% indicates partial engagement, and ≤40% indicates poor engagement. These engagement levels will be correlated with outcomes to assess intervention effectiveness. To evaluate external validity, a feedback scale will be used to collect qualitative data from participants. This scale will focus on how the intervention translates into their daily lives, covering aspects such as satisfaction with social functioning, academic or job performance, and other relevant real-world outcomes.

### Analysis

#### Effectiveness Analyses

The primary hypothesis for the between-group difference in the primary outcome will be tested using a linear mixed-effects model. This model accounts for responses at postintervention and the 3-month follow-up, incorporating baseline outcome measures and intervention assignment as fixed effects, with a participant-specific random intercept. An interaction between time and the randomized group will also be fitted as a fixed effect to allow estimation of treatment effect at all time points. A similar approach will be used for the secondary outcome analysis. The imputation of missing values will be based on the assumption that data are missing at random. The primary analyses will follow the intention-to-treat principle, meaning participants will be analyzed based on their originally assigned groups, regardless of their adherence to entry criteria, the treatment received, or any withdrawal or deviation from the protocol [39]. The data will be automatically generated by the WeChat miniprogram once participants complete the process, eliminating the need for manual data entry. A *P* value <.05 will be used as the level of statistical significance. Data analysis will involve at least 2 statisticians or researchers and will be processed and verified using various statistical software, such as SAS and SPSS.

#### Cost-Effectiveness Analyses

Quality-adjusted life years (QALYs) will be used for economic evaluation to compare the relative costs and outcomes of different groups. Estimating quality of life weights involves quantifying the quality of life at different health states on a scale from 0, representing the worst imaginable health state or death, to 1, indicating perfect health. These weights are directly measured in this study population using standardized instruments (ie, EQ-5D-5L). To calculate QALYs, the quality of life weights are multiplied by the duration spent in each health state, with the results summed over the study period. Direct health care utilization, such as outpatient visits and prescribed medications, will be recorded, while indirect costs are excluded because most expected participants are secondary- or early tertiary-level students without a full-time income. Incremental cost-effectiveness ratios will then be calculated as the difference in mean cost divided by the difference in mean QALYs between the OkeyMind and waiting list groups.

## Results

The study was funded in April 2024, and the first participant was enrolled in September 2024; as of the end of March 2025, 132 participants had been recruited. Recruitment is ongoing. Data collection will conclude in July 2025, after which data analysis will begin and be completed in August 2025, with primary findings targeted for publication in spring 2026. The study’s design, management, analysis, and reporting are independent of the funder.

Demographic features, such as age, gender, and education level, will be reported to provide a comprehensive overview of the participant population. The primary analysis, focusing on social anxiety, will be conducted using an intention-to-treat approach, carefully accounting for missing values. Various visualizations, including trend graphs, charts, flow diagrams, and other illustrative tools, will be used to depict participants’ changes over time. Additionally, subgroup analyses will be included to explore variations within specific segments of the participant population.

Metrics such as intensity and frequency of use will also be reported to offer insights into participants’ engagement with the intervention. Last, any significant changes in the software or delivery of the intervention during the study period will be documented and reported to ensure transparency and to contextualize the findings.

## Discussion

### Expected Findings

The anticipated outcomes of this study aim to contribute to and expand upon the existing literature on mHealth interventions, particularly for youths with social anxiety. One expected finding is an improvement of access to mental health support. Prior studies have demonstrated that mHealth interventions improve accessibility for populations facing geographical, financial, or social barriers to traditional therapy [10]. This research builds on these findings by evaluating a WeChat-integrated intervention, which leverages a widely used platform to facilitate low-barrier engagement for youth with social anxiety. Another expected outcome is the provision of empirical evidence for effectively addressing social anxiety in youth. Early intervention of social anxiety is crucial to prevent long-term impairment. This study contributes to filling that gap by evaluating a mobile psychoeducation intervention designed to be culturally embedded, accessible, and engaging for young individuals. By leveraging mobile technology, this research advances empirical understanding of scalable, youth-centered mHealth interventions for social anxiety. Finally, the study anticipates economic and cost-effectiveness benefits. Previous research suggests that mHealth interventions can be cost-effective, particularly when reducing the demand for in-person services and optimizing health care resource allocation [19,20]. This study extends those findings by incorporating a cost-analysis component, providing concrete data on the economic impact of mobile-based psychoeducation for social anxiety, which remains underexplored in the current literature.

### Limitations

Given that all intervention sessions and evaluations are conducted online and the research period extended over several months, there is potential for a relatively high attrition rate compared with on-site interventions. To address this issue, several measures are implemented. First, during the screening stage, interviews are scheduled to ensure each participant has the proper motivation and desire to participate, helping to select committed individuals more likely to complete the program. Second, support is provided during interventions and assessments, with manpower allocated to address any issues. Reminder systems, such as texts or calls, are implemented to prompt participants when delays occur, aiming to keep them engaged and on track. Third, the technology and software team continuously improves the user experience based on participant feedback, enhancing video quality and reducing buffering times to ensure a smoother and more enjoyable experience, thus reducing dropout rates due to technical frustrations.

Additionally, the technological nature of OkeyMind may limit the program’s generalizability. Youths familiar with using social media and WeChat miniprograms are likely to benefit more from the intervention than older adults who may be less familiar and comfortable with these digital platforms. This discrepancy in technological proficiency could impact the overall effectiveness and reach of the program across different age groups, potentially introducing bias in the results and affecting the external validity of the study. Future research should consider addressing this limitation by exploring ways to make the program more accessible and user-friendly for older adults.

Last, achieving complete participant blinding could be challenging, particularly in behavioral interventions such as this study. Participants could infer their group assignment, introducing potential performance and self-report biases. However, using a waiting list rather than a no-treatment control ensured that control participants still anticipated full access to the intervention after follow-up, potentially softening, though not eliminating, these biases. Future studies should adopt more rigorous designs, such as concurrently running 2 active intervention arms, to mitigate performance and self-report biases.

### Conclusions

In conclusion, this research aims to evaluate the effectiveness and cost-effectiveness of a mobile-based psychoeducation program, OkeyMind, designed to address social anxiety among youth. OkeyMind represents an innovative and promising strategy to combat social anxiety, especially in low- and middle-income countries where medical resources are limited. By leveraging mobile technology, this program has the potential to provide accessible and cost-effective mental health support to a population that might otherwise lack adequate resources, thereby improving mental health outcomes on a broader scale.

## Abbreviations

CONSORT: Consolidated Standards of Reporting Trials
mHealth: mobile health
PHQ-9: Patient Health Questionnaire-9
QALYs: quality-adjusted life years
SPIN: Social Phobia Inventory

## Data Availability

De-identified data are available upon reasonable request to the corresponding author.
